# BiDRA: Bayesian inference as a robust alternative to non-linear regression for dose–response efficiency metrics assessment

**DOI:** 10.1093/bioadv/vbag245

**Published:** 2026-08-22

**Authors:** Caroline Labelle, Petr Smirnov, Maud David, Mario Callejo, Benjamin Haibe-Kains, Anne Marinier, Sébastien Lemieux

**Affiliations:** Institute for Research in Immunology and Cancer, Université de Montréal, Montréal, Québec, H3T 1J4, Canada; Princess Margaret Cancer Centre, University Health Network, Toronto, Ontario, M5G 2M9, Canada; Medical Biophysics, University of Toronto, Toronto, Ontario, M5G 1L7, Canada; Institute for Research in Immunology and Cancer, Université de Montréal, Montréal, Québec, H3T 1J4, Canada; Institute for Research in Immunology and Cancer, Université de Montréal, Montréal, Québec, H3T 1J4, Canada; Princess Margaret Cancer Centre, University Health Network, Toronto, Ontario, M5G 2M9, Canada; Medical Biophysics, University of Toronto, Toronto, Ontario, M5G 1L7, Canada; Vector Institute, Toronto, Ontario, M5G 0C6, Canada; Ontario Institute for Cancer Research, Toronto, Ontario, M5G 0A3, Canada; Department of Computer Science, University of Toronto, Toronto, Ontario, M5S 1A1, Canada; Dalla Lana School of Public Health, University of Toronto, Toronto, Ontario, M5T 3M7, Canada; Institute for Research in Immunology and Cancer, Université de Montréal, Montréal, Québec, H3T 1J4, Canada; Department of Chemistry, Université de Montréal, Montréal, Québec, H3C 3J7, Canada; Institute for Research in Immunology and Cancer, Université de Montréal, Montréal, Québec, H3T 1J4, Canada; Department of Biochemistry and Molecular Medicine, Université de Montréal, Montréal, Québec, H3C 3J7, Canada; Mila - Quebec AI Institute, Montréal, Québec, H2S 3H1, Canada

## Abstract

**Motivation:**

Dose–response metrics such as the half-maximal inhibitory concentration, high-dose–response, and slope are central to drug discovery, yet standard Levenberg–Marquardt fits often produce biased or unsupported values for incomplete curves and lack uncertainty measures. In practice, this forces experimenters to visually inspect each curve fit to judge its reliability, a tedious, subjective, and non-scalable process.

**Results:**

Across 421 405 public dose–response experiments from three large pharmacogenomic datasets, shared-concentration viability responses were highly consistent across biological replicates, making it reasonable to expect derived efficiency metrics to show comparable replicate behavior when supported by the data. However, Levenberg–Marquardt fits to incomplete curves often produced unsupported metric values, including inflated rates of observable potency estimates (>70% versus ∼39% complete curves), which could falsely suggest metric-level disagreement between similar replicate responses. BiDRA represents efficacy, potency, and slope as posterior distributions, allowing uncertainty to remain large when the data do not support precise metric inference. This avoids treating uncertainty-dominated responses as conflicting point estimates, exposes unsupported Levenberg–Marquardt estimates, and supports uncertainty-aware compound ranking. We illustrate its utility in a structure–activity relationship screen, demonstrating how posteriors enable robust, criteria-based selection in drug discovery settings.

**Availability and implementation:**

BiDRA is implemented in Julia and available at https://github.com/lemieux-lab/bidra_robustness.

## 1 Introduction

Large-scale dose–response screens are used to test the efficacy of therapeutic agents across biological contexts (Haibe-Kains *et al.* 2013). Compound efficacy is commonly summarized using metrics such as the half maximal effective/inhibitory concentration (EC_50_/IC_50_), high- and low-dose–responses (HDR and LDR), and slope (Neubig *et al.* 2003). These metrics are typically estimated from experimental responses using non-linear regression, most commonly through the Levenberg–Marquardt algorithm (Levenberg 1944, Marquardt 1963). However, this point-estimation approach has important limitations. It relies only on observed responses and provides no direct measure of uncertainty, even though incomplete or unresponsive curves can represent a substantial fraction of large screening datasets (Rahman *et al.* 2019). As a result, fitted efficiency metrics can be unreliable or unsupported by the experimental data. Experimenters often compensate using pre- and post-analysis interventions, such as filtering, capping, or visual inspection, but these approaches are often subjective and can increase analytical variability (Simeone and Costa 2022). Bayesian inference (Gelman *et al.* 2013, Lambert 2018) directly addresses this limitation by representing efficiency metrics as posterior distributions rather than single point estimates. BiDRA (Labelle *et al.* 2019) and other Bayesian dose–response models (Huson and Kinnersley 2009, Hennessey *et al.* 2010, Johnstone *et al.* 2016, Simeone and Costa 2022, Tansey *et al.* 2022, Zhang *et al.* 2023) have previously been proposed to infer such posteriors, but their robustness relative to standard Levenberg–Marquardt fitting has not yet been quantified systematically across large pharmacogenomic datasets.

Using three large publicly available datasets [Gray (Heiser *et al.* 2012, Daemen *et al.* 2013), gCSI (Errington *et al.* 2014, Klijn *et al.* 2015) and CTRPv2 (Basu *et al.* 2013, Seashore-Ludlow *et al.* 2015, Rees *et al.* 2016), Fig. 1A], we quantitatively compared Levenberg–Marquardt fitting and Bayesian inference for estimating dose–response efficiency metrics across experimental contexts. We also present a revised version of BiDRA (Labelle *et al.* 2019) (Bayesian inference for Dose–Response Analysis), with updated priors designed to improve robustness to common dose–response fitting issues. Our analyses quantify the limitations of Levenberg–Marquardt fitting, particularly for incomplete or unresponsive curves, and show that posterior-based representations distinguish data-supported metric values from uncertainty-dominated cases. This distinction is important because single-point estimates can turn unsupported fits into apparently precise metric values, thereby creating or amplifying apparent discrepancies between replicate experiments (Haibe-Kains *et al.* 2013, Safikhani *et al.* 2016b). Finally, we demonstrate how posterior distributions can support compound selection in a structure-activity relationship (SAR) study by propagating uncertainty into ranking, comparison, and selection criteria.

**Figure 1:**
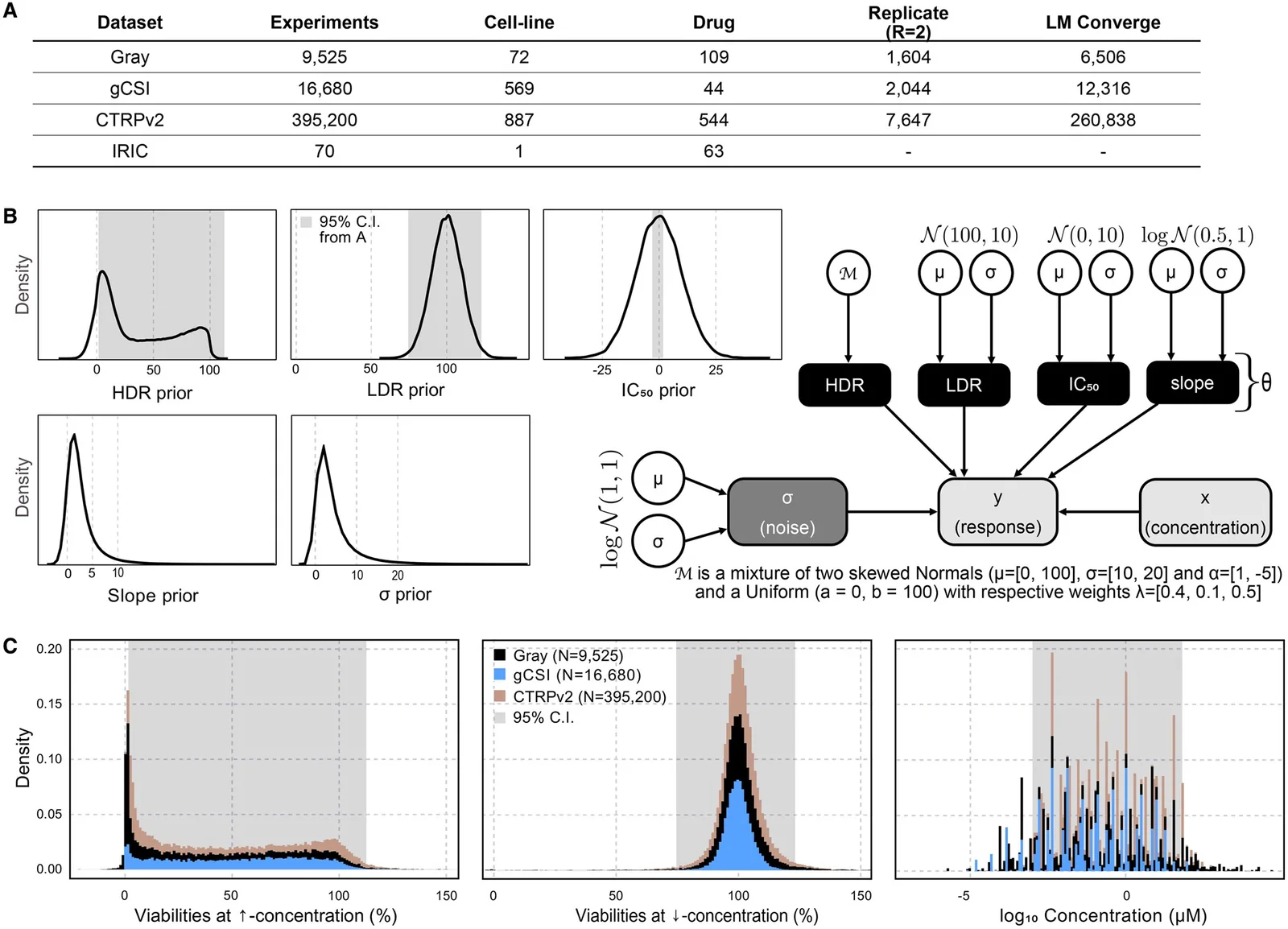
Datasets and Bayesian model overview. (A) Dataset descriptions and number of unique drugs, cell lines, and dose–response experiments. (B) Prior distributions and schematic representation of the BiDRA Bayesian model. Shaded regions in the HDR, LDR, and IC_50_ prior densities correspond to the empirical intervals shown in C. In the BiDRA model schematic, prior distribution parameters are represented by circles, efficiency metrics by black boxes, the likelihood variance by a gray box, and experimental data by light-gray boxes. (C) Distributions of responses measured at the highest (↑) and lowest (↓) tested concentrations, and of experimental concentrations, across the Gray, gCSI, and CTRPv2 datasets (421 405 experiments). Shaded regions represent the central 95% empirical intervals, also shown in B.

## 2 Methods

### 2.1 Data

For our robustness demonstration, we considered three large publicly available datasets [Gray (Heiser *et al.* 2012, Daemen *et al.* 2013), gCSI (Errington *et al.* 2014, Klijn *et al.* 2015) and CTRPv2 (Basu *et al.* 2013, Seashore-Ludlow *et al.* 2015, Rees *et al.* 2016)], and for our *posterior*s application demonstration, we used a smaller in-house dose–response screen (IRIC; Fig. 1A). Dataset composition, replicate definitions, data retrieval, and preprocessing steps (including filtering thresholds and normalization procedures) are detailed in Note 4, available as supplementary data at *Bioinformatics Advances* online.

### 2.2 Compound efficacy metrics

#### 2.2.1 Dose–response model

We used the standard 4-parameter log–logistic model to describe dose–response curves (1); parameter definitions are in Note 5, available as supplementary data at *Bioinformatics Advances* online.


(1) $$f(x,\theta )=\mathrm{HDR}+\frac{\mathrm{LDR}-\mathrm{HDR}}{1+10^{\mathrm{slope}\cdot (x-IC_{50})}}$$

Levenberg–Marquardt fits were performed in Julia using LsqFit.jl with default settings (full implementation in Supp. Note 6).

#### 2.2.2 BiDRA: Inferring Bayesian posteriors of efficiency metrics

BiDRA is a hierarchical Bayesian 4-parameter log–logistic model (Fig. 1B) fit with NUTS (Turing.jl; Labelle *et al.* 2019). Weakly informative *priors* reflect plausible experimental ranges (full specification in Supp. Notes 7 and 9). Implementation code is available at https://github.com/lemieux-lab/bidra_robustness.

Briefly, Bayesian inference for Dose–Response Analysis [BiDRA (Labelle *et al.* 2019)] is a hierarchical Bayesian model (Fig. 1B) that infers *posteriors* of efficiency metrics (θ). Similar to Levenberg–Marquardt, BiDRA assumes that experimental responses are normally distributed around f(x, θ) with a shared standard deviation (SD, σ, 2).


(2) $$\begin{array}{c} P(\theta |y)\propto P(y|\theta )\cdot P(\theta )\\ P(y|\theta )∼N(f(x,\theta ),\sigma^{2}) \end{array}$$

More formally, the probability of θ given some experimental response y is proportional to the product of the likelihood of observing said response given θ, and θ *prio*rs. We sampled four chains of 1000 retained iterations each, yielding 4000 posterior samples per efficiency metric and experiment. We refer to these distributions as *posteriors*.

#### 2.2.3 Inference sensitivity to priors

To assess our model sensitivity to the chosen *priors* (full specification in Supp. Notes 7 and 9), we used synthetic response sets. The latter were generated by sampling n responses from a known dose–response curve (LDR = 100.0, HDR = 0.0, log_10_ IC_50_ = 0.0 and slope = 1.0) and adding random Gaussian noise (σ = 1.0 and σ = 10.0). To assess possible variation in sensitivity to the priors, 100 response sets were generated for each level of noisiness (Fig. 10A, available as supplementary data at *Bioinformatics Advances* online). BiDRA was then used to infer efficiency metrics with a sequentially decreasing number of data points, starting at the highest concentrations (Fig. 10B and C, available as supplementary data at *Bioinformatics Advances* online). To show the role played by the priors within BiDRA’s, metrics were also inferred using a Bayesian model with uninformative uniform *priors* (Fig. 10B and C, available as supplementary data at *Bioinformatics Advances* online), all bound between −1000 and 1000 [with the exception of σ ∼ U(0.001, 1000)]. Other inference parameters were the same as for BiDRA.

### 2.3 Replicate-wise comparison of efficiency metric representations

#### 2.3.1 Correlation coefficients

As there is no consensus on the metric to use (Safikhani *et al.* 2016b, Rahman *et al.* 2019), we used Pearson and Spearman rank correlation coefficients to summarize replicate-wise relationships between efficiency metric representations. Pearson correlation measures linear co-variation and is sensitive to outliers, whereas Spearman rank correlation measures monotonic co-variation by calculating Pearson correlation on ranked values. Because correlation does not directly measure agreement, we interpreted these coefficients as descriptive summaries of replicate-wise co-variation rather than as measures of concordance.

The assignment of paired replicates to the two correlation axes is arbitrary, and independently reversing the orientation of individual pairs can affect the resulting coefficient. To remove dependence on this convention, we computed orientation-averaged Pearson and Spearman rank correlation coefficients. For each analysis, replicate orientation was independently randomized within each pair over 10 000 repetitions, and the resulting correlation coefficients were averaged. Pearson correlation was used as the primary analysis, while Spearman rank correlation was used as a sensitivity analysis.

#### 2.3.2 Correlation between responses of biological replicates

We first considered correlation between responses of pairs of biological replicates (Fig. 2). The aim of this analysis was to establish if we could expect some correlation between their efficiency metrics, as the latter are derived from individually measured responses. RMSΔ computation and per-dataset pairing details are in Supp. Note 8.

#### 2.3.3 Replicate-wise correlation of Levenberg–Marquardt point estimates

We considered 1604, 2044, and 7647 pairs of biological replicates for Gray, gCSI, and CTRPv2, respectively. For each efficiency metric (LDR, HDR, IC50, and slope), we computed the orientation-averaged Pearson and Spearman rank correlation coefficients defined above (Fig. 3). We performed these analyses for three groups of biological replicate pairs: (i) all pairs, (ii) pairs in which both experiments had complete dose–response curves (SD ≥ 20, see *Response Set Completeness* section below), and (iii) pairs in which both experiments had incomplete or unresponsive dose–response curves (SD < 20).

#### 2.3.4 Replicate-wise correlation of BiDRA posterior quantiles

Each posterior was summarized using 15 evenly spaced quantiles at the midpoints of 15 equal-width probability intervals, $\left\{\frac{j-0.5}{15},:\;\mathrm{for}\;j=1,\ldots ,15\right\}$, including the posterior median (50th quantile). At each quantile, every experiment therefore contributed a single quantile-specific value. Using the same biological replicate pairs and response-completeness groups as for the Levenberg–Marquardt analysis, we computed orientation-averaged Pearson and Spearman rank correlation coefficients separately for each posterior quantile (Fig. 3).

This quantile-specific approach provides a distribution-wide view of replicate-wise correlation while preserving the distinction between different regions of the posterior distribution. Unlike calculating a single correlation from all posterior samples, it gives each experiment one value per quantile and therefore prevents the number of posterior samples from determining the effective sample size of the correlation analysis. The median correlation corresponds to the 50th quantile, while variation across the remaining quantiles reflects how replicate-wise correlation changes across the posterior distributions.

#### 2.3.5 Random-pair control experiment

To provide a negative baseline for the replicate-wise correlation analysis, we generated random pairs by retaining the first replicate of each biological pair and randomly permuting the second replicates across pairs. This procedure preserved the number of pairs and the marginal composition of the two replicate sets while disrupting their original drug-cell-line correspondence. We then applied the same analysis as for the true biological pairs, computing orientation-averaged Pearson and Spearman rank correlation coefficients for Levenberg–Marquardt point estimates and for each of the BiDRA posterior quantile-specific summaries. Analyses were performed for all randomly paired experiments, pairs in which both responses were complete (SD ≥ 20), and pairs in which both responses were incomplete or unresponsive (SD < 20). Results are presented in Fig. 6A and B, available as supplementary data at *Bioinformatics Advances* online.

### 2.4 Robustness evaluation

To evaluate how curve completeness affected metric estimation and inference, we compared response completeness with Levenberg–Marquardt point estimates and BiDRA posterior distributions for IC_50_ and HDR (Fig. 4).

#### 2.4.1 Response set completeness

We expected replicate-wise metric behavior to depend in part on curve completeness and therefore required a practical metric to characterize completeness. We used the SD of the response values as a pragmatic proxy for curve completeness. Small SD values are generally associated with incomplete or unresponsive curves, whereas larger SD values are generally associated with complete curves. Visually, curve completeness is typically assessed from the presence or absence of both plateaus and from the number of observations forming the high-concentration plateau.

Based on visual inspection of multiple response sets, we selected an SD threshold of 20. We therefore classified experiments with SD < 20 as incomplete or unresponsive, and experiments with SD ≥ 20 as complete (Fig. 4A). We used this classification to compare how Levenberg–Marquardt point estimates and BiDRA posterior distributions behaved across complete and incomplete response curves (Fig. 4C–E).

#### 2.4.2 IC_50_ and HDR

Curve completeness affects metric estimation and inference. We focus our analysis on the IC_50_ and HDR, two metrics commonly used to characterize compound potency and efficacy, respectively. LDR is generally less affected by curve completeness because it represents the basal response at low concentrations.

To assess whether a Levenberg–Marquardt IC_50_ estimate was observable within the tested concentration range, we compared it with the experiment-specific concentration range, denoted by D (Fig. 4C). Estimates were classified as observable when IC_50_ ∈ D and unobservable otherwise. For BiDRA posteriors, we calculated the posterior probability that IC_50_ lay outside the experimental concentration range, P(IC_50_ ∉ D; Fig. 4D).

To quantify posterior uncertainty, we calculated the width of the 95% credible interval, denoted by Δ. We selected this measure because its interpretation was more direct than that of the posterior SD or median absolute deviation.

### 2.5 Application of BiDRA to SAR analysis and compound selection

The analysis and interpretation of multiple posterior distributions can be nontrivial for experimenters. We therefore demonstrate how posterior distributions can be used to rank compounds and evaluate selection criteria while propagating uncertainty.

#### 2.5.1 Compound ranking

Given N compounds and their *posterior distributions for a* metric *θ_i_*, we estimated the posterior probability that each compound occupied each possible rank. At each of the 4000 posterior sampling positions, one draw from each compound posterior was ranked across compounds in ascending or descending order, depending on the metric. The empirical frequency with which compound $C_{j}$ occupied rank r was then used to estimate its rank probability,


(3) $$\mathrm{Pr}(R_{j}=r|\theta_{i}),\;r\in \{1,\ldots ,N\}.$$

This procedure yielded a posterior rank probability distribution for each compound, which we represented as a heatmap. For visualization, compounds were ordered according to their posterior median values (Fig. 5A). We identified compounds with a probability of at least *p* of occupying one of the first *R* ranks:


(4) $$\sum_{r=1}^{R}\mathrm{Pr}R_{j}=r\;|\;\theta_{i})\geq p.$$

#### 2.5.2 DAG representation

We used a directed acyclic graphs (DAGs) to summarize pairwise relationships among a set of *N* compounds (Fig. 5B). To construct the graph, we compared every ordered pair of compounds as described in our initial paper (Labelle *et al.* 2019). For each ordered pair ($C_{i}$, $C_{j}$), we estimated the posterior probability that $C_{i}$ was preferable to $C_{j}$ for the metric of interest. For IC_50_, a directed edge from $C_{i}$ to $C_{j}$ was added when


(5) $$\mathrm{Pr}({{\mathrm{IC}}_{50}}_{i}<{{\mathrm{IC}}_{50}}_{j})\geq 1-\alpha ,$$

indicating that $C_{i}$ was more potent than $C_{j}$ at the selected posterior probability threshold. Redundant edges were then removed by transitive reduction (Aho *et al.* 1972). The probability was estimated empirically from the proportion of the 4000 paired posterior draws satisfying the inequality.

#### 2.5.3 Compound selection

The ranking and DAG approaches described above facilitate visualization of compound relationships and support compound selection. They help identify compounds that are most likely to occupy the preferred ranks and compounds that meet a specified posterior probability threshold for improvement over a reference compound. Both approaches can also be combined with additional selection criteria, as illustrated by the SAR analysis in Fig. 5. For a criterion defined on an efficiency metric, we estimated the posterior probability of meeting that criterion as the proportion of posterior draws satisfying it. We combined the marginal criterion probabilities multiplicatively to obtain a global selection score. These posterior-based analyses propagate metric uncertainty into ranking, pairwise comparison, and compound selection, reducing the reliance on unsupported point estimates and subjective filtering.

## 3 Results

### 3.1 BiDRA model and priors

*Priors* play an important role in BiDRA’s inference, as they allow the experimenter to mathematically incorporate prior knowledge regarding the experimental context (Chen *et al.* 1999, Albert *et al.* 2012). *Priors* are soft constraints on the possible values for each metric. When the observed responses do not support precise inference for a metric, such as when the high-dose plateau is missing, the posterior remains broad and prior-influenced rather than collapsing to an unsupported point estimate. Our sensitivity analysis demonstrates the constraining action of the *priors* and supports their suitability in the specific context of dose–response experiments (Fig. 10B and C, available as supplementary data at *Bioinformatics Advances* online). When we first introduced BiDRA (Labelle *et al.* 2019), we demonstrated the importance of using appropriate, weakly informative *priors*. We revisited and redefined our *priors* to better represent the plausible range of responses and concentrations (Fig. 1B). The HDR and LDR priors were supported via a visual inspection of the distributions of viability responses for the highest (HDR) and lowest (LDR) doses over the datasets (Fig. 1C). Similarly, the wide range of experimental concentrations (Fig. 1C) and the difficulty of constraining the range of possible values for the IC_50_ justified the use of a wide and weakly informative *prior* for this metric (Fig. 1B). *Priors* were not mathematically fitted to the experimental data distributions but were defined through *prior* elicitation.

### 3.2 Response-level consistency across replicates

Response-level consistency across biological replicates was assessed as a baseline indicator of reproducibility. When numerically comparing shared-concentration responses from biological replicates (Fig. 2A–C), we found that all three datasets had similar viability responses across replicates, with their respective Pearson correlation coefficients being above or close to 0.75 (Gray: 0.86, gCSI: 0.88 and CTRPv2: 0.74; Fig. 2D). Furthermore, most individual pairs of replicates had a root mean square of response differences (RMSΔ) consistent with well-replicated response values (RMSΔ < 20, Fig. 2E). Only 0.5% of all pairs considered shared a single concentration (54 pairs in gCSI, Fig. 2A), making it difficult to evaluate consistency. Pairs with a large number of shared concentrations generally had a lower RMSΔ, further supporting response-level consistency across replicates (Fig. 2A–C). These results establish that the observed viability responses are generally reproducible across replicates. Therefore, apparent discrepancies among downstream efficiency metrics should be interpreted in light of how each method represents metric values when the underlying dose–responses do not provide sufficient support for precise metric inference.

**Figure 2:**
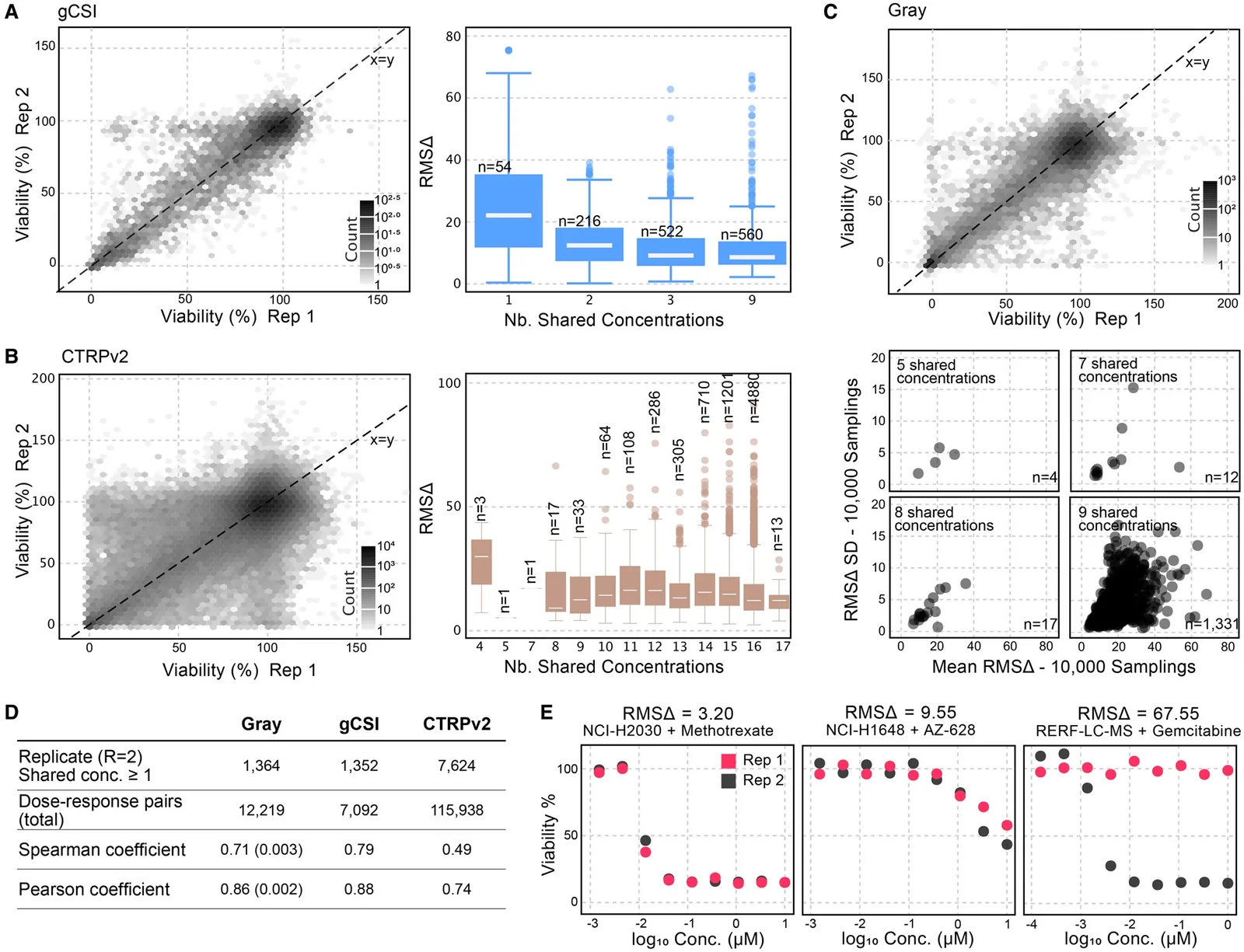
Response consistency across biological replicates. Hexbin plots in (A–C) compare viability responses measured at shared concentrations across biological replicates. Boxplots in A and B show the distribution of RMSΔ values according to the number of shared concentrations. The number of biological replicate pairs in each group is denoted by *n*. (A) Response consistency assessment for gCSI. (B) Response consistency assessment for CTRPv2. (C) Response consistency assessment for Gray. The hexbin plot shows one random pairing of within-concentration replicates. The four lower panels show the mean and SD of RMSΔ values across 10 000 random samplings and pairings, stratified by the number of shared concentrations. (D) Summary of replicate counts and response-level correlation coefficients by dataset. For Gray, correlation coefficients are reported as the mean (SD) across 10 000 random samplings and pairings of within-concentration replicates. (E) Examples of biological replicate pairs from gCSI with low (RMSΔ = 3.20), intermediate (RMSΔ = 9.55), and high (RMSΔ = 67.55) response differences.

### 3.3 Replicate-wise comparison of efficiency metrics

We next compared efficiency metric representations across biological replicates using Levenberg–Marquardt point estimates and BiDRA posterior quantile-specific summaries. Comparisons were performed for all replicate pairs and for subsets defined by response completeness: All Pairs, Complete Pairs, and Incomplete Pairs (Fig. 3 and Fig. 1, available as supplementary data at *Bioinformatics Advances* online). We used SD of the response values to group experiments: small SD values (< 20) are characteristic of seemingly unresponsive (eg PF-4708671 in Fig. 4A) or incomplete curves (eg AZ-628 and Lapatinib in Fig. 4A), whereas larger SD (≥ 20) are generally indicative of complete curves (Gemcitabine in Fig. 4A).

**Figure 3:**
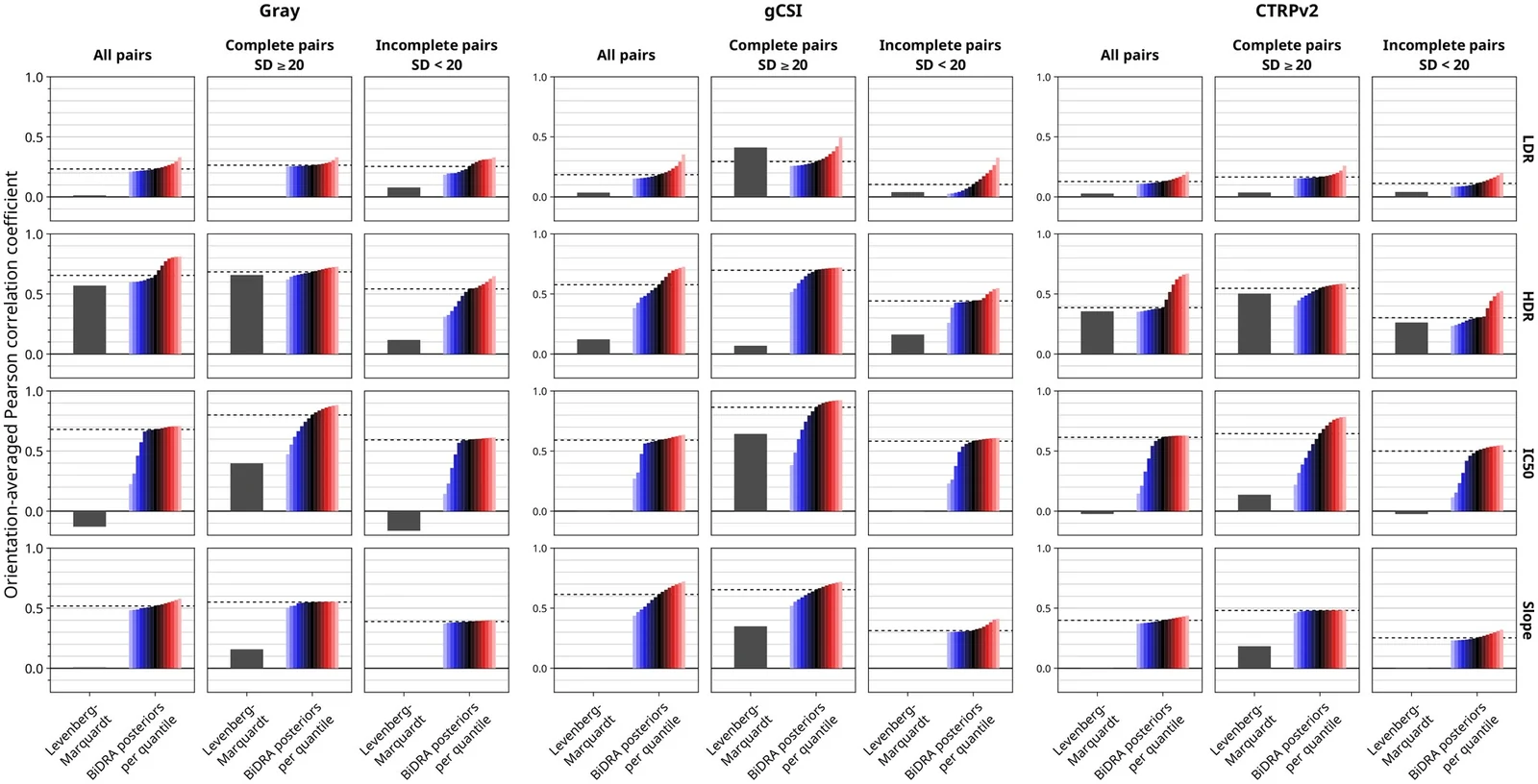
Replicate-wise correlations of efficiency metric representations. Orientation-averaged Pearson correlation coefficients are shown across efficiency metrics (rows), datasets (column triplets), and replicate-pair groupings (columns). Gray bars represent Levenberg–Marquardt point estimates, whereas colored bars represent the 15 BiDRA posterior quantile-specific summaries, ordered from lower to upper quantiles. Dashed horizontal lines indicate the correlation obtained from the BiDRA posterior median, and solid horizontal lines indicate zero correlation. Replicate-pair groupings were defined using response SD as a proxy for curve completeness. Complete Pairs contain two experiments with SD ≥ 20, whereas Incomplete Pairs contain two experiments with SD < 20.

**Figure 4:**
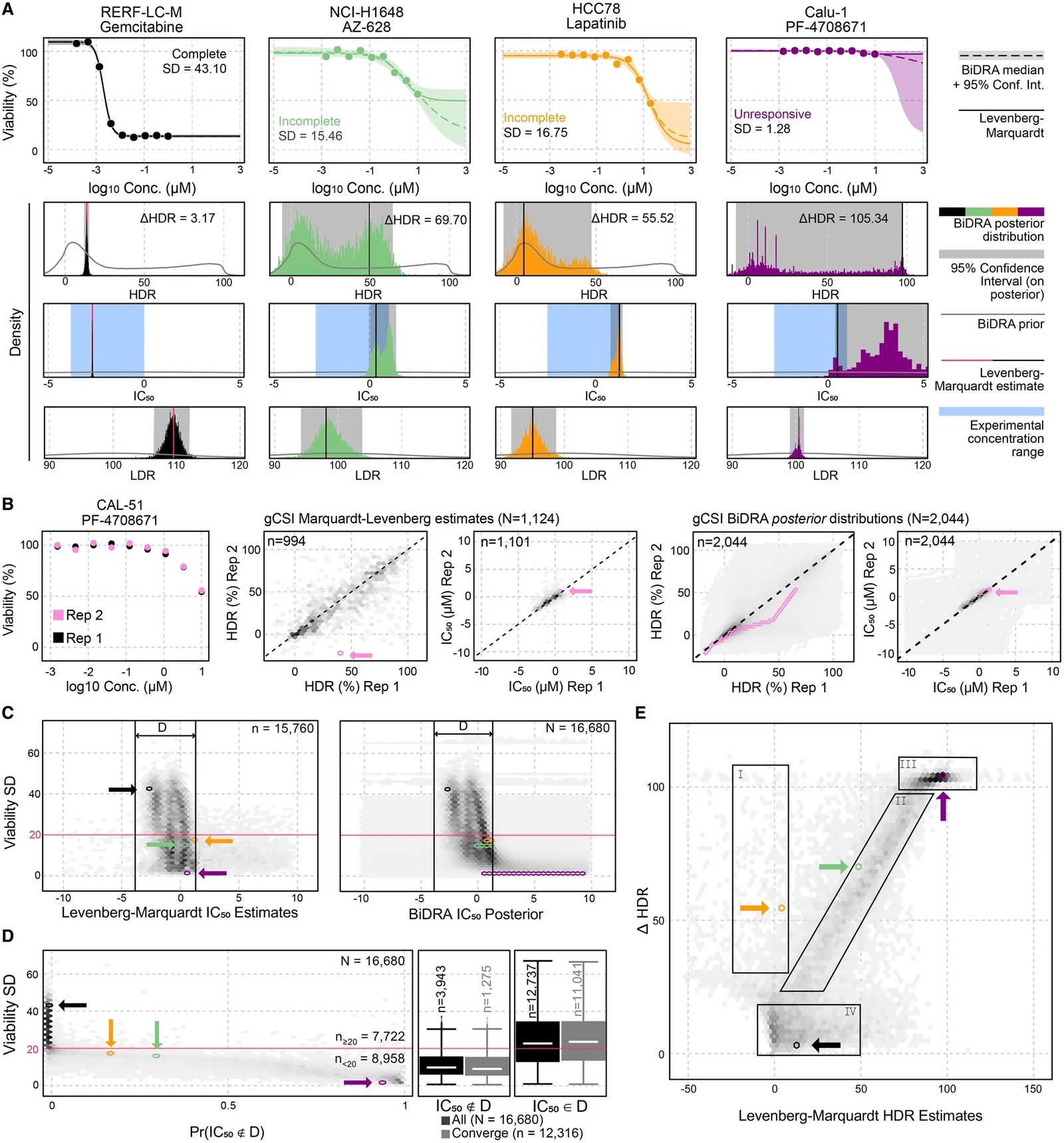
BiDRA’s robustness to incomplete and unresponsive dose–response curves. (A) Experiments with different response-completeness classifications (Complete, Incomplete, and Unresponsive), characterized using response SD. Levenberg–Marquardt fitted curves and BiDRA posterior median curves with 95% credible intervals are shown. Levenberg–Marquardt point estimates and BiDRA posterior distributions for HDR, IC_50_, and LDR are displayed below each response curve. The same experiments are highlighted using the corresponding colors in C–E. (B) PF-4708671 biological replicate pair with similar observed responses. Levenberg–Marquardt HDR and IC_50_ point estimates and paired BiDRA posterior quantiles for the highlighted pair (pink) are shown relative to the corresponding gCSI biological replicate pairs. Dashed lines represent the identity diagonal. (C) Levenberg–Marquardt IC_50_ point estimates and BiDRA IC_50_ posterior samples plotted against response SD. Black vertical lines delimit the 95% interval of tested concentrations, denoted by D. The total number of experiments and the number in highlighted subsets are denoted by N and n, respectively. (D) (Left) Relationship between response SD and the posterior probability that IC50 lies outside the experimental concentration range, $Pr(IC_{50}\notin D)$. (Right) Distribution of response SD for observable and unobservable Levenberg–Marquardt IC_50_ estimates, stratified by convergence status. Numbers of experiments are denoted by N for all experiments and n for subsets. (E) Relationship between Levenberg–Marquardt HDR point estimates and BiDRA posterior width, quantified as the width of the 95% credible interval (ΔHDR). Regions I–IV highlight representative combinations of point estimates and posterior uncertainty discussed in the main text. Panels C–E show results for gCSI; corresponding analyses for Gray and CTRPv2 are presented in Fig. 7, available as supplementary data at *Bioinformatics Advances* online.

To remove dependence on arbitrary replicate orientation, we computed an orientation-averaged Pearson correlation coefficient. For each replicate pair, replicate orientation was randomly swapped independently across 10 000 repetitions, and the resulting coefficients were averaged. We also computed an orientation-averaged Spearman rank correlation coefficient as a sensitivity analysis (Fig. 1, available as supplementary data at *Bioinformatics Advances* online).

Across datasets and metrics, Levenberg–Marquardt point estimates and BiDRA posterior quantile-specific summaries showed distinct orientation-averaged correlation patterns. For Pearson correlations (Fig. 3), BiDRA posterior summaries generally showed higher correlations than Levenberg–Marquardt point estimates, particularly for HDR, IC50, and slope. However, the Spearman sensitivity analysis (Fig. 1, available as supplementary data at *Bioinformatics Advances* online) showed a more nuanced pattern: for LDR and HDR, BiDRA posterior summaries were often comparable to Levenberg–Marquardt estimates, whereas the reduced apparent discrepancy for incomplete pairs was maintained most clearly for IC50 and slope. The generally weak LDR correlations can be explained by the limited variability of LDR values across experiments (Fig. 2, available as supplementary data at *Bioinformatics Advances* online). For All Pairs, the posterior median correlation was higher than the Levenberg–Marquardt point-estimate correlation for HDR, IC50, and slope in most datasets, while the full set of posterior quantiles showed how this correlation varied across the posterior distribution.

The All Pairs coefficients should be interpreted in light of the Complete-to-Incomplete pair ratio in each dataset. For Complete Pairs, where the response data provide stronger support for precise metric inference, Levenberg–Marquardt point estimates and BiDRA posterior quantile-specific summaries showed more similar behavior. The largest differences were observed for Incomplete Pairs. In these cases, Levenberg–Marquardt often returned specific point estimates despite limited support from the observed responses, leading to weak replicate-wise correlations, especially for IC50 and slope. In contrast, BiDRA posterior quantiles frequently retained positive correlations because both replicates remained similarly uncertainty-dominated when the data did not support precise metric inference.

Consequently, these positive correlations among posterior quantiles should not be interpreted as precise agreement on a metric value. Rather, they indicate that posterior-based representations preserve uncertainty instead of forcing unsupported metrics into specific point estimates that may appear discrepant across replicates.

Finally, random-pair controls provided a negative baseline for the replicate-wise correlation analysis. When second replicates were randomly permuted across pairs, orientation-averaged Pearson and Spearman correlations were close to zero across datasets, metrics, response-completeness subsets, and BiDRA posterior quantiles (Note 1, available as supplementary data at *Bioinformatics Advances* online; Fig. 6A and B, available as supplementary data at *Bioinformatics Advances* online). Thus, while priors can influence individual posteriors when responses are incomplete, the positive correlations observed among true replicate pairs are not explained by shared priors alone.

### 3.4 BiDRA’s robustness to incomplete responses

The results presented in Fig. 3 suggest that the main differences between the two methodologies arise from how they represent efficiency metrics when dose–response curves are incomplete or unresponsive (Fig. 4A). The PF-4708671 pair (Fig. 4B) illustrates how posterior distributions avoid overinterpreting incomplete dose–response curves. Levenberg–Marquardt IC_50_ estimates differed between replicates (0.69 μM for Rep1 and 1.21 μM for Rep2), but BiDRA’s IC_50_ posteriors indicate that this difference should be interpreted in light of their uncertainties. Levenberg–Marquardt HDR estimates were poorly supported by the observed responses and differed substantially between replicates (41.65% for Rep1 and −18.39% for Rep2), which could incorrectly suggest a replicate discrepancy even though the observed viabilities are nearly identical. In contrast, BiDRA returned wide HDR posteriors for both replicates, indicating that both HDRs were highly uncertain and that the observed data do not support calling the replicate HDR values discrepant.

Algorithmically, Levenberg–Marquardt forces a single fitted curve onto the observed responses, which can result in unreliable efficiency metric estimates when the response does not support all parameters of the model. For instance, we observed that Levenberg–Marquardt frequently estimated observable IC_50_ values, meaning that the estimated concentration was within the experimental concentration range (denoted by D in Fig. 4C and Fig. 7, available as supplementary data at *Bioinformatics Advances* online). This likely overestimates the true number of experimentally supported observable IC_50_ values, as illustrated by the estimated IC_50_ for Calu-1 + PF-4708671 (purple in Fig. 4C). Indeed, in gCSI, for instance, 12 737 IC_50_ estimates (76.36% of experiments) were within the experimental concentration range, whereas only 6501 responses (38.97%) were classified as complete (SD ≥ 20). Although an incomplete response can be informative regarding the approximate IC_50_ location, the resulting point estimate may still be poorly supported. We observed similar distributions when comparing viability SD to observed and unobserved IC_50_ estimates, regardless of Levenberg–Marquardt convergence status (Fig. 4D and Fig. 7, available as supplementary data at *Bioinformatics Advances* online). In contrast, incomplete responses had a higher probability of having IC_50_  *posterior* mass outside of the experimental concentration range [Pr(IC_50_ ∉ D) in Fig. 4D and Fig. 7, available as supplementary data at *Bioinformatics Advances* online].

BiDRA represents uncertainty in efficiency metrics by returning wider posterior distributions when the data provide limited support for precise inference, as demonstrated by the comparisons of ΔHDR to Levenberg–Marquardt estimated HDR (Fig. 4E and Fig. 7, available as supplementary data at *Bioinformatics Advances* online) and to response completeness (SD values, Fig. 8, available as supplementary data at *Bioinformatics Advances* online). We identified many experiments with highly uncertain HDR *posteriors* and likely unsupported Levenberg–Marquardt estimates close to the optimal 0% (orange and I in Fig. 4E). We also observed that higher HDR estimates were often associated with wider HDR posteriors (green and II in Fig. 4E). This group most likely corresponds to incomplete dose–response curves. We could also identify seemingly unresponsive curves for which Levenberg–Marquardt returned a specific HDR estimate close to 100% despite the absence of support from the observed responses (purple and III in Fig. 4E). For complete curves, both methodologies generally yielded similar HDR values, with BiDRA returning narrow posteriors consistent with well-supported metric inference (black and IV in Fig. 4E).

### 3.5 Post-inference analysis: compound selection

Given the IRIC dataset (Fig. 1A), collected as part of a hit-to-lead optimization project, we asked whether any synthesized analogs improved over the initial reference compound. We define improvement as higher efficacy, corresponding to large HDR, and greater potency, corresponding to smaller EC_50_, relative to the reference compound. We applied BiDRA to the 70 dose–response experiments and collected *posteriors* for each of the standard efficiency metrics: LDR, HDR, EC_50_, and Slope. We used these posterior representations to perform compound selection while propagating uncertainty in each efficiency metric.

From the EC_50_ rank probabilities, we identified 16 experiments with a probability of at least 0.90 of being among the first 20 ranks (purple in Fig. 5A). Of these, 10 are Analogs and 6 are replicates of the Reference. Overall, several experiments could be probabilistically distinguished in terms of EC_50_. We also noticed subgroups of compounds (eg Analogs 58 and 46) with similar ranking probabilities, suggesting similar EC_50_ posteriors and indicating that the data do not support a precise ordering within these subgroups. Replicates of the Reference had EC_50_ posteriors with similar rank probability distributions (blue in Fig. 5). The slight shifts in rank probabilities most likely reflect differences in EC_50_ posterior width and location, illustrating how uncertainty in the inferred metric is propagated into the ranking procedure. The EC_50_ and HDR posteriors of Reference replicates mostly overlap, except for Reference B, which had a higher HDR posterior (Fig. 5A). The globally low and uncertain HDR observed was characteristic of the cell line used and was expected. Further experiments were done to enhance HDR while maintaining potency but are not shown here.

**Figure 5:**
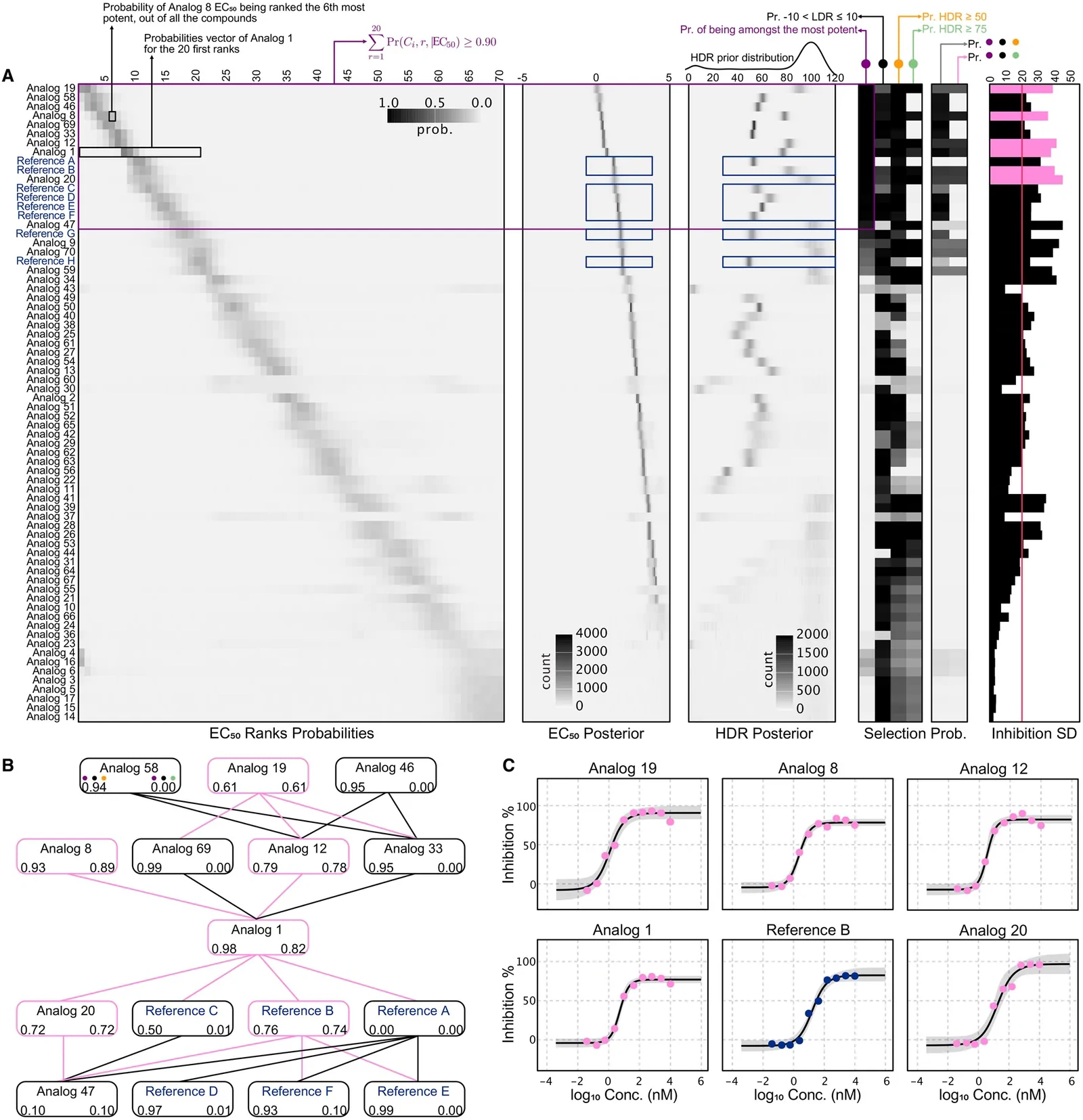
SAR analysis and compound selection using posterior distributions. (A) EC_50_ rank probabilities and posterior-based selection criteria. From left to right: (1) posterior probabilities of each EC_50_ rank, with experiments ordered by increasing EC_50_ posterior median; experiments with a probability of at least 0.90 of occupying one of the first 20 ranks are enclosed by the purple rectangle; (2) EC_50_ posterior distributions, ordered as in (1); (3) HDR posterior distributions, ordered as in (1); (4) posterior probabilities of satisfying the individual potency, LDR, and HDR selection criteria, with larger probabilities shown in black; and (5) combined selection scores for the two sets of criteria. Response SD, used as a proxy for curve completeness, is shown in the rightmost bar plot. Experiments displayed in C are highlighted in pink, with the Reference shown in blue. (B) DAG summarizing pairwise EC_50_ comparisons among the most potent subset identified in A. A directed path indicates a statistically supported difference in potency at α=0.05; redundant edges were removed by transitive reduction. The two combined selection scores are reported within each node. (C) Posterior median dose–response curves for the experiments selected from A. Shaded regions represent 95% credible intervals.

We used a DAG to summarize statistically supported EC_50_ comparisons (⍺ = 0.05) from our 16-experiment subset (Fig. 5). If there exists a path between two nodes of DAG, then the EC_50_ posterior of the more potent experiment is statistically supported as lower than that of the less potent experiment at ⍺ = 0.05. For instance, Analog 58 was statistically supported as more potent than Analog 12.

We computed, for each experiment, the probability of meeting each selection criterion (Fig. 5A): (potency) The experiment must be among the most potent, ie its EC_50_ must have a probability of at least 0.90 of being among the first 20 ranks; (LDR ≈ 0) the experiment must have a basal response (LDR) between −10 and 10, to ensure quality control over the normalization of the response; (HDR ≥ 50) the experiment must have high efficacy, ie its HDR must be at least 50%; (HDR ≥ 75) the experiment must have high efficacy, ie its HDR must be at least 75%. We obtained global selection scores by combining, for each experiment, the probabilities of meeting the individual criteria (Fig. 5A). The two global probabilities differ in terms of the high-efficacy criterion (HDR). Because we were interested in potent compounds with high efficacy, we used the second global selection score (potency, LDR ≈ 0, HDR ≥ 75). We identified six potent, high-efficacy compounds with complete dose–response curves (SD ≥ 20; highlighted in Fig. 5; dose–response curves in Fig. 5C). Based on these criteria, Analogs 1, 8, 12, and 19 were identified as better than the Reference.

## 4 Discussion

Dose–response analyses often guide costly compound-selection decisions (Carnero 2006, Berdigaliyev and Aljofan 2020), making uncertainty-aware efficiency metrics critical. Although widely used (Naqa *et al.* 2006, Gadagkar and Call 2015, Veroli *et al.* 2015, Clark *et al.* 2017), Levenberg–Marquardt fitting has important disadvantages, namely its dependence on the number and placement of experimental concentrations and data points (Seefeldt *et al.* 1995, Rahman *et al.* 2019), and the lack of a direct measure of estimate uncertainty (Simeone and Costa 2022). Across three large datasets, these shortcomings were most apparent for incomplete or flat responses (further discussed in Supp. Note 10). For complete, well-defined sigmoidal responses (SD ≥ 20), the algorithm can provide well-supported efficiency metric estimates. When responses are either incomplete or flat (SD < 20), efficiency metric estimates are often extreme and poorly supported by the experimental data. This behavior is illustrated by our assessment of IC_50_ observability: most Levenberg–Marquardt IC_50_ estimates fell within the experimental dose range, even when the response profile did not support a complete curve (Fig. 4C and Fig. 7, available as supplementary data at *Bioinformatics Advances* online).

Due to the lack of a direct statistical measure of estimate uncertainty (Newman 2008, Simeone and Costa 2022), experimenters must rely on goodness-of-fit metrics [such as the square root of the mean of the squared residuals, RMSE (Labelle *et al.* 2019)], convergence status, or qualitative inspection of the fitted dose–response curve. Unfortunately, goodness-of-fit is not necessarily indicative of whether a metric estimate is supported by the observed response (Labelle *et al.* 2019, Simeone and Costa 2022), and neither is convergence status (Fig. 4D and Fig. 7, available as supplementary data at *Bioinformatics Advances* online). Visual inspection (Bouhaddou *et al.* 2016) is also a poor alternative, as it is experimenter-specific, difficult to reproduce, and tedious to carry out at scale. The apparent plausibility of efficiency metric estimates can also be enhanced through data manipulation, such as dataset filtering(Safikhani *et al.* 2016a; eg filtering out flat-response experiments), experimental response capping (eg responses cannot exceed 100%) and estimated efficiency metrics values capping (Smirnov *et al.* 2018, Feizi *et al.* 2022) [e.g. capping IC_50_ values to the largest experimental dose (Rahman *et al.* 2019)]. These interventions are subjective, difficult to reproduce at scale, and can increase analytical variability or remove informative but incomplete responses(Rahman *et al.* 2019).

BiDRA addresses these cases by representing each efficiency metric as a posterior distribution rather than a single fitted value. This representation combines experimental responses with weakly informative *priors*, allowing posterior uncertainty to remain large when the data do not support a precise estimate. It also enables post-inference analyses that propagate uncertainty into decision-making. Here, we proposed an updated version of our BiDRA hierarchical model (Labelle *et al.* 2019; Fig. 1B). We defined weakly informative *priors* for all parameters through *prior* elicitation (Fig. 1B). The *prior* distributions were calibrated to reflect our collaborators’ expert opinions and to encompass at least 95% of the available experimental data (ie the central 95% empirical intervals across > 420k experiments, shaded regions in Fig. 1B and C). This calibration allows the likelihood to dominate when responses are informative, while letting priors act as soft constraints when specific metric values are not well supported by the experimental responses (Fig. 10B and C, available as supplementary data at *Bioinformatics Advances* online). Entirely removing informative structure from the *priors* (see uniform priors in Fig. 10B and C, available as supplementary data at *Bioinformatics Advances* online) impaired Bayesian sampling, likely because the sampler had little posterior structure to guide exploration.

We used a normal sampling distribution (likelihood) to mirror the least-squares behavior of Levenberg–Marquardt fitting. We also retained the four-parameter log-logistic model rather than using less-parameterized versions of the model (Hennessey *et al.* 2010, Johnstone *et al.* 2016). While less-parameterized models can be appropriate in some experimental contexts (Johnstone *et al.* 2016), we found that they can be too constraining and can have an effect similar to an overly narrow *prior* (Labelle *et al.* 2019). We therefore recommend inferring all efficiency metrics, as each can provide complementary information about compound sensitivity (Fallahi-Sichani *et al.* 2013, Rahman *et al.* 2019). For instance, a wide LDR *posterior* can flag possibly improper response normalization arising from problematic negative controls and can be used for quality control assessment (Fig. 5A). We also demonstrated how the *posterior* distributions of basic efficiency metrics can be used to derive other efficiency metrics, such as the area above the curve (Haibe-Kains *et al.* 2013, Smirnov *et al.* 2018; AAC, Note 2, available as supplementary data at *Bioinformatics Advances* online and Figs 4 and 5, available as supplementary data at *Bioinformatics Advances* online) or DSS (Yadav *et al.* 2014).

A central contribution of this work is the large-scale replicate-based benchmark of uncertainty-aware efficiency metric representations. Unlike previous qualitative or simulation-based demonstrations (Hennessey *et al.* 2010, Johnstone *et al.* 2016, Labelle *et al.* 2019, Simeone and Costa 2022), this benchmark compares how Levenberg–Marquardt point estimates and BiDRA posterior quantile-specific summaries behave across biological replicates in three large datasets. We used the orientation-averaged Pearson correlation coefficient as the primary replicate-wise correlation metric and treated the orientation-averaged Spearman rank correlation coefficient as a sensitivity analysis. Because correlation is not agreement, these coefficients were interpreted as descriptive summaries of replicate-wise co-variation, not as direct measures of concordance. The Pearson analysis showed that BiDRA posterior summaries generally had higher replicate-wise correlations than Levenberg–Marquardt point estimates, particularly for IC_50_ and slope (Fig. 3). The Spearman sensitivity analysis showed a more nuanced pattern: BiDRA posterior summaries were often comparable to Levenberg–Marquardt estimates for LDR and HDR, whereas the reduction in apparent discrepancy for incomplete pairs was maintained most clearly for IC_50_ and slope (Fig. 1, available as supplementary data at *Bioinformatics Advances* online).

The largest methodological differences were observed for incomplete or unresponsive responses (SD < 20). In these cases, Levenberg–Marquardt often returned specific point estimates despite limited support from the observed responses, leading to weak replicate-wise correlations, especially for IC_50_ and slope. In contrast, BiDRA posterior quantiles often retained positive correlations consistent with both replicates being similarly uncertainty-dominated when the data did not support precise metric inference. These positive correlations should not be interpreted as agreement on a precise metric value. Rather, they indicate that posterior-based representations preserve uncertainty instead of forcing unsupported metrics into specific point estimates that may appear discrepant across replicates. Random-pair controls further supported this interpretation. When experiments were paired independently of drug and cell line, orientation-averaged Pearson and Spearman correlations were close to zero across datasets, metrics, response-completeness subsets, and BiDRA posterior quantiles (Note 1, available as supplementary data at *Bioinformatics Advances* online; Fig. 6A and B, available as supplementary data at *Bioinformatics Advances* online). Thus, while priors can influence individual posteriors when responses are incomplete, the positive correlations observed among true replicate pairs are not explained by shared priors alone.

Another novel element of this work is our demonstration of an end-to-end post-inference analysis for compound selection from a SAR screen (Fig. 5). However, the interpretation and practical use of posterior distributions can be nontrivial. When developing a novel methodology, it is important to demonstrate both its accessibility and utility (Wheeler *et al.* 2023). Using the IRIC dataset, we highlighted several post-inference analyses, including efficiency metric ranking, experiment comparisons, and evaluation of selection criteria. These analyses accounted for both experimental and analytical uncertainty, considered the entire dataset regardless of response completeness, and provided statistically supported comparisons, ranking and combined selection scores. These analyses directly depend on posterior distributions and are therefore not available from Levenberg–Marquardt point estimates alone.

This study has a few limitations that define the scope within which BiDRA’s output should be interpreted. Our results apply primarily to dose–response experiments for which a four-parameter log-logistic model provides a plausible representation of the underlying response. BiDRA is not intended to make unsupported efficiency metrics precise; rather, its main contribution is to make explicit when the available experimental data do not support precise inference. This distinction is particularly important for incomplete or unresponsive curves, where the posterior may be strongly influenced by weakly informative priors because key regions of the curve, such as the high-dose plateau or the IC_50_ transition, are only partially observed or not observed at all. In such cases, some metrics—most notably HDR when the high-dose plateau is missing—may remain intrinsically uncertain. The proposed priors were deliberately chosen to be broad and were evaluated across several large pharmacogenomic datasets and an in-house SAR screen, but applications involving markedly different response scales, concentration ranges, assay readouts, or non-sigmoidal behavior may require prior re-elicitation and model checking before interpretation.

BiDRA also trades computational efficiency for uncertainty quantification (Fig. 11, available as supplementary data at *Bioinformatics Advances* online). In our implementation, the runtime for Bayesian posterior inference was more than 1000-fold longer than for Levenberg–Marquardt fitting. This relative difference is substantial, but the absolute runtime remained modest, at approximately one second per dose–response experiment on four CPU cores. For most pharmacological screens, this cost is unlikely to dominate the overall analysis workflow, although it may become relevant for very large-scale analyses or when computational resources are limited.

Finally, discrepancies within and across large-scale pharmacogenomic datasets should be revisited by considering *posterior* distributions as efficiency metric representations. Previous studies have emphasized biological and technical variability, including the challenge of replicating experiments across laboratories and research centers (Niepel *et al.* 2019). Our results suggest that the representation of efficiency metrics is also an important driver of apparent discrepancies: point estimates can turn unsupported fits into specific values that appear discrepant across experiments, whereas posterior distributions preserve the underlying uncertainty. This perspective may facilitate the integration of dose–response datasets while reducing reliance on subjective curve filtering, metric capping, or manual exclusion of inconclusive fits.

## Supplementary Material

vbag245_Supplementary_Data

## Data Availability

The Gray, gCSI and CTRPv2 datasets can be downloaded from PharmacoDB (Feizi *et al*. 2022)/ORCESTRA (Mammoliti *et al*. 2021) and handled using PharmacoGx (Smirnov *et al*. 2016). The IRIC dataset can be made available on request. Our code is accessible at https://github.com/lemieux-lab/bidra_robustness.
