# Supplementary material for: BiDRA: Bayesian inference as a robust alternative to non-linear regression for dose–response efficiency metrics assessment
Source: Bioinform Adv. 2026 Aug 22;6(1):vbag245. doi: 10.1093/bioadv/vbag245 (PMC13626134; doi:10.1093/bioadv/vbag245)
Supplement: Supplementary file 1 [file vbag245_supplementary_data.docx]

**BiDRA: Bayesian Inference as a Robust Alternative to Non-Linear Regression for Dose-Response Efficiency Metrics Assessment**

Caroline Labelle, Petr Smirnov, Maud David, Mario Callejo, Benjamin Haibe-Kains, Anne Marinier, Sébastien Lemieux

[Supplementary Text	2](#_Toc228980404)

[Supp. Note 1. Control experiments	2](#_Toc228980405)

[Supp. Note 2. Area Above the Curve (AAC) consistency across replicates	2](#_Toc228980406)

[Supp. Note 3. Other definitions of replicates	4](#_Toc228980407)

[Supp. Note 4. Dataset composition, replicate definitions, data retrieval, and preprocessing steps	4](#_Toc228980408)

[Supp. Note 5. Dose–response model	5](#_Toc228980409)

[Supp. Note 6. Levenberg-Marquardt implementation	6](#_Toc228980410)

[Supp. Note 7. BiDRA model’s details and implementation	6](#_Toc228980411)

[Supp. Note 8. Correlation between responses of biological replicates	8](#_Toc228980412)

[Supp. Note 9. BiDRA’s *priors’* definition and their impact on the inference	9](#_Toc228980413)

[Supp. Note 10. Estimating efficiency metrics of incomplete response sets	11](#_Toc228980414)

[Supplementary Figures	13](#_Toc228980415)

[Supp. Figure 1. Efficiency metrics consistency across replicates	13](#_Toc228980416)

[Supp. Figure 2. Concordance of the asymptotic basal responses between biological replicates	15](#_Toc228980417)

[Supp. Figure 3. Assessment of common experimental dose for pairs of biological replicates from the gCSI dataset	16](#_Toc228980418)

[Supp. Figure 4. Concordance of area above the curve (AAC) between biological replicates	17](#_Toc228980419)

[Supp. Figure 5. Exploring special cases of AAC calculation for Levenberg-Marquardt and BiDRA	19](#_Toc228980420)

[Supp. Figure 6. Spearman’s rank correlations for control experiments	21](#_Toc228980421)

[Supp. Figure 7. Comparison of IC50 and HDR estimates and their posterior for Gray and CTRPv2	22](#_Toc228980422)

[Supp. Figure 8. Comparison of HDR estimates and posterior uncertainty to curve completeness	23](#_Toc228980423)

[Supp. Figure 9. Assessment of efficiency metrics correlations for within dataset multi-replicates and across datasets singletons	24](#_Toc228980424)

[Supp. Figure 10. Illustrative examples of the effects of missing datapoints on curve fittings approaches and of Bayesian models’ sensitivity to their *priors*	26](#_Toc228980425)

[Supp. Figure 11. Comparison of Levenberg-Marquardt’s and BiDRA’s computational efficiency	28](#_Toc228980426)

[References	29](#_Toc228980427)

# Supplementary Text

Supp. Note 1. Control experiments

To provide a negative baseline for the replicate-wise correlation analysis, we disrupted the biological pairing structure by retaining the first replicate of each pair and randomly permuting the second replicates across pairs. This procedure preserved the number and marginal composition of the two replicate sets while disrupting their original (drug, cell-line) correspondence.

We then applied the same analysis used for true biological replicate pairs. Orientation-averaged Pearson and Spearman rank correlation coefficients were computed for Levenberg-Marquardt point estimates and for each of the 15 BiDRA posterior quantile-specific summaries. Analyses were performed for all randomized pairs, pairs in which both experiments had complete responses (SD ≥ 20), and pairs in which both experiments had incomplete or unresponsive responses (SD < 20).

For both Pearson and Spearman analyses, correlations were close to zero across datasets, efficiency metrics, response-completeness groups, and posterior quantiles (Supp. Fig. 6A–B). Thus, although weakly informative priors can influence individual posteriors when the data provide limited support for precise metric inference, shared priors alone did not induce positive correlations between unrelated experiments. The positive correlations observed among true biological replicate pairs therefore reflect information preserved by the original replicate pairing rather than an artifact of the posterior representation or shared priors.

Supp. Note 2. Replicate-wise comparison of Area Above the Curve (AAC)

We calculated AAC values using LDR as the baseline from Levenberg-Marquardt point estimates and from each BiDRA posterior draw. For BiDRA, this yielded a posterior distribution of AAC for each experiment. Because AAC depends on the integration range, we first determined the common experimental concentration range for each pair of biological replicates (Supp. Fig. 3). The distributions of these common ranges confirmed that they were generally sufficient for AAC calculation (Supp. Fig. 3C). We then compared Levenberg-Marquardt AAC point estimates and BiDRA AAC posterior medians using the same orientation-averaged Pearson and Spearman rank correlation analyses and response-completeness groupings as for the basic efficiency metrics (Supp. Figs. 4–5).

BiDRA AAC posterior medians generally showed higher orientation-averaged correlations than Levenberg-Marquardt AAC point estimates. Differences were modest for Complete Pairs and larger for Incomplete Pairs (Supp. Figs. 4-5). As for the basic efficiency metrics, these higher correlations should not be interpreted as direct evidence of agreement. Rather, the posterior-based AAC representation preserves uncertainty when the observed responses do not support a precise curve-derived summary.

Supp. Note 3. Other definitions of replicates

We defined biological replicates in the primary analyses as (cell-line,compound) combinations represented by exactly two experiments within a single dataset (R = 2). This restriction ensured that each combination contributed a single replicate pair. We excluded combinations represented by at least three experiments within a dataset (R ≥ 3) and experiments corresponding to the same combination across different datasets.

We quantified the prevalence of alternative within-dataset replicate structures (Supp. Fig. 9A–B). Gray contained 2,283 singletons (R = 1), 1,604 combinations with R = 2, and 1,135 combinations with R ≥ 3. The corresponding counts were 10,597, 2,044, and 529 for gCSI, and 378,587, 7,647, and 437 for CTRPv2. Combinations with R ≥ 3 were therefore relatively frequent in Gray but uncommon in gCSI and CTRPv2. In all three datasets, most combinations with R ≥ 3 were represented by three experiments (Supp. Fig. 9A).

We also counted within-dataset singletons corresponding to the same (cell-line,compound) combination in two datasets. Gray shared 6 such combinations with gCSI and 26 with CTRPv2, whereas gCSI and CTRPv2 shared 4,025 combinations (Supp. Fig. 9B). These matched experiments were not included as biological replicates because they were generated in different datasets and therefore potentially differed in their experimental and technical conditions.

Supp. Note 4. Dataset composition, replicate definitions, data retrieval, and preprocessing

Gray, gCSI, and CTRPv2 differ in their coverage of cancer cell lines and compounds and contain varying numbers of experiments for individual (cell-line,compound) combinations (Fig. 1A). We defined biological replicates as experiments in which the same compound was tested in the same cell line within a single dataset. For the primary analyses, we considered combinations represented by exactly two experiments (R = 2), such that each combination contributed one replicate pair. We excluded combinations represented by more than two experiments (R ≥ 3) and matched experiments from different datasets. The prevalence of these alternative replicate structures is reported in Supplementary Figure 9.

The Gray, gCSI and CTRPv2 datasets were downloaded from PharmacoDB^1^/ORCESTRA^2^ and processed using PharmacoGx^3^. The retrieved normalized responses represent cell viability, expressed as a percentage and decreasing with increasing dose. Concentrations were expressed in μM and log_10_-transformed. We excluded experiments with at least one response greater than 200% or lower than –50%. After preprocessing, we retained 9,525, 16,680 and 395,200 individual dose-response experiments (i.e., unique dose-response curves) from Gray, gCSI, and CTRPv2, respectively (Fig. 1A).

The IRIC screen is a subset of a larger structure-activity relationship (SAR) study designed to identify analog compounds with high efficacy and greater potency than a reference compound. The subset analyzed here contains 70 dose-response experiments performed in a single leukemia cell line. In total, 62 analog compounds and one reference compound were tested (Fig. 1A). Cell responses were quantified using a luminescence assay. Luminescence values were normalized using the mean luminescence of negative controls (DMSO-treated cells) located in the same plate row. The normalized responses represent cell-growth inhibition, expressed as a percentage and increasing with increasing dose..

Supp. Note 5. Dose–response model

We use the four-parameter log-logistic model^4^ to describe the experimental dose-response relationship:

$$f\left( x, \theta\right) = HDR + \frac{LDR - HDR}{1 + {10}^{slope\cdot\left( x - {IC}_{50} \right)}}$$

( 1 )

In Equation (1), $x$ denotes log_10_-transformed experimental concentration, and $f\left( x, \theta\right)$ denotes the expected response for a given set of parameters θ. The four free parameters $\theta=\left( LDR,HDR,IC_{50},slope \right)$, are hereafter referred to as efficiency metrics.

**LDR** and **HDR**: The two asymptotic plateaus of the dose–response curve, corresponding to the expected responses at low and high doses, respectively. Their interpretation depends on the type of response analyzed. For a decreasing dose-response relationship, LDR represents the upper response plateau, whereas HDR represents the lower response plateau.

**IC_50_**: The concentration corresponding to the inflexion points of the dose-response curve, at which the expected response lies midway between LDR and HDR. Because concentration were log_10_-transformed, IC_50_ values are also expressed on the log_10_ scale. For simplicity, we refer to this parameter as IC_50_. For the ascending dose-response curves in the IRIC dataset, we refer to the corresponding parameter as EC_50_.

**Slope**: The parameter controlling the steepness of the dose-response curve around its inflection point. This parameter is often referred to as the Hill slope or Hill coefficient.

For our primary analyses, we only considered these four efficiency metrics. Alternative summary metrics, including the area under or above the curve^5^ (AUC/AAC) and drug sensitivity score^6^ (DSS), can also be derived from fitted or sampled θs.

Supp. Note 6. Levenberg-Marquardt implementation

We estimated efficiency metrics through non-linear regression. To do so, we used the Levenberg-Marquardt algorithm^7,8^ as implemented by LsqFit.jl [https://github.com/JuliaNLSolvers/LsqFit.jl]. We initialized θ = {LDR, HDR, IC_50_, slope} with p_0_ = [100, 0, 0, 1] and used a maximum of 1,000 iterations. Because the algorithm can converge before reaching this limit, we recorded whether convergence was achieved for each experiment (Fig. 1A).

We estimated efficiency metrics independently for each experiment in Gray, gCSI and CTRPv2. From an estimated parameter vector θ_LM_, a dose-response curve can be generated by evaluating $f(x,\theta_{LM})$ over a selected range of concentrations. Examples are shown as solid lines in Figure 4A. The corresponding θ_LM_ values are indicated by the black and red vertical lines in the metrics subplots. We refer to the efficiency metrics obtained with this approach as Levenberg-Marquardt estimates.

Supp. Note 7. BiDRA model’s details and implementation

BiDRA (Bayesian inference for Dose-Response Analysis^9^) is a hierarchical Bayesian model (Fig. 1B) that infers *posteriors* of efficiency metrics θ. Similarly to Levenberg-Marquardt, BiDRA assumes that experimental responses are normally distributed around $f\left( x, \theta\right)$ with a shared standard deviation (σ) across observations of the same experiment:

$$P\left( \theta|y \right)\propto P\left( y|\theta\right)\cdot P\left( \theta\right)$$

$$P\left( y|\theta\right)\mathcal{\sim N}\left( f\left( x,\theta\right), \sigma^{2} \right)$$

Thus, the posterior distribution of θ combines the likelihood of the observed responses with prior information about the efficiency metrics. Unlike Levenberg-Marquardt regression, which yields one point estimate for each metric, BiDRA produces posterior samples representing the range and relative support of parameter values given the data and the model.

BiDRA uses the No-U-Turn sampler (NUTS)^10^, a Hamiltonian Monte Carlo algorithm. For each experiment, we ran four independent Markov chain Monte Carlo chains of 2,000 iterations. The first 1,000 iterations of each chain were discarded as warmup, and the remaining 1,000 iterations were retained, yielding 4,000 posterior samples per efficiency metric and experiment. We set the target acceptance probability $\delta$ to 0.65. During warmup, NUTS adapted its step size to approach this target. Relative to the default value of $\delta=0.8$, this setting generally permits larger integration steps and reduced the computational burden of analyzing more than 420,000 experiments.

For this study, BiDRA was implemented in Julia using Turing.jl [https://turing.ml/stable/] and MCMCChains.jl [https://github.com/TuringLang/MCMCChains.jl] with source code available at https://github.com/lemieux-lab/bidra_robustness. We inferred the posterior distributions of the efficiency metrics independently for each experiment in Gray, gCSI, CTRPv2, and IRIC.

Prior distributions encode information about the model parameters before considering the response values from an individual experiment. We selected weakly informative priors intended to constrain the parameters to experimentally plausible ranges without overwhelming the information supplied by the observed responses. The priors used for the decreasing dose-response curves in Gray, gCSI, and CTRPv2 were (Supp. Note 9):

- $HDR\mathcal{\sim M}$where $\mathcal{M}$ is a mixture of two skewed Normal (μ = [0, 10], σ = [10, 20] and ⍺= [1, -5]) and a $\mathcal{U}(0, 100)$ with respective weights 𝜆 = [0.4, 0.1, 0.5]
- $LDR \sim\mathcal{N}(100,10)$
- ${IC}_{50} \sim\mathcal{N}(0,10)$
- $slope \sim log \mathcal{N}(0.5,1)$
- $\sigma\sim log \mathcal{N}(1,1)$

For the increasing dose-response curves in the IRIC dataset, the LDR and HDR priors were adjusted to reflect the corresponding response scale and curve orientation.

The prior distributions were selected to reflect the experimental context and are illustrated in Figure 1C. Responses measured at the lowest concentrations generally showed limited variation around the basal response, supporting a relatively informative LDR prior. By contrast, responses measured at the highest concentrations varied broadly, primarily between 0% and 100%, motivating the wider HDR prior. The response distributions shown in Figure 1C were used to illustrate the plausibility of the priors rather than to estimate their parameters. The same priors were shared across experiments and public datasets. Log-normal priors were used for slope and σ to constrain these parameters to positive values. Because the asymptotes are defined according to dose as LDR and HDR rather than according to their numerical values as minimum and maximum responses, the slope parameter can remain positive for both decreasing and increasing curves.

Posterior samples of $\theta$ can also be propagated through the dose-response function to summarize uncertainty in the expected response curve. For each retained posterior draw $i$, we evaluated $f\left( x,\theta_{i} \right)$ over a selected range of hypothetical concentrations. This produced a posterior distribution of the expected response at each concentration. We then calculated the pointwise posterior median and the 2.5^th^ and 97.5^th^ percentiles, yielding a pointwise 95% credible interval. Alternatively, individual curves generated from posterior draws can be plotted to display the distribution of dose-response relationships directly.

Examples of pointwise posterior median curves and their 95% credible intervals are shown in Figure 4A. The corresponding efficiency-metric posteriors are displayed as colored histograms, together with the prior densities in light gray and the Levenberg-Marquardt estimates as black or red vertical lines.

Supp. Note 8. Response-level comparison of biological replicates

Replicated experiments do not always share the same concentrations. We limited our analysis to the comparison of shared-concentration responses (Fig. 2). Most replicate pairs shared at least one concentration and could therefore be compared: 84% in Gray, 66% in gCSI and 99% in CTRPv2. Most comparable pairs in Gray and CTRPv2 shared at least four and five concentrations, respectively. In gCSI, 3% of replicate pairs shared only one concentration. Nevertheless, most comparable pairs in all three datasets shared responses at multiple concentrations.

We characterized response-level co-variation using Pearson and Spearman rank correlation coefficients and directly quantified response differences using the root mean square of response differences (RMSΔ):

$RMS\Delta= \sqrt{\sum_{i=1}^{N} \frac{\left( y_{1i}-y_{2i} \right)^{2}}{N}}$	(1)

Here, $N$ is the number of shared concentrations between two replicated experiments, and $y_{1i}$ and $y_{2i}$ are the responses of the two experiments at the $i$^th^ shared concentration. The designation of the experiments as the first or second replicate is arbitrary and does not affect RMSΔ. A lower RMSΔ indicates more similar response values at shared concentrations, whereas a higher RMSΔ indicates larger response differences (Fig. 2E). RMSΔ estimates based on only one or a few shared concentrations should be interpreted with greater caution. However, most replicate pairs shared multiple concentrations, allowing RMSΔ to summarize response-level consistency across several measurements.

The gCSI and CTRPv2 datasets contained one response value per experiment at each concentration. Thus, each shared concentration provided one pair of response values for comparison (Fig. 2A-B). We analyzed 1,352 gCSI and 7,624 CTRPv2 replicate pairs, corresponding to 7,092 and 115,938 shared-concentration response pairs, respectively.

Gray experiments contained multiple response measurements at each concentration. For each concentration shared by two replicated experiments, we randomly selected and paired one response from each experiment. We calculated RMSΔ from the resulting response pairs, repeated this procedure 10,000 times, and reported the mean and standard deviation of RMSΔ across repetitions (Fig. 2C). We analyzed 1,364 Gray replicate pairs, encompassing 12,219 shared-concentration response measurements.

Supp. Note 9. Definition of BiDRA priors and their impact on inference

Priors are fundamental to Bayesian inference and represent uncertainty about model parameters before considering the observations from a given experiment. Bayes' theorem combines the prior distribution of θ with the likelihood of the observed responses to obtain its posterior distribution (Eq. 2, main text). In BiDRA, priors act as soft constraints that assign greater probability to plausible parameter values while retaining support over broad ranges. Their influence is greater when the experimental responses provide little information about a parameter, whereas sufficiently informative responses dominate the posterior inference. Priors therefore allow experimental knowledge and context to be incorporated into the model while limiting support for biologically implausible values. For example, a response of -3,500% would be highly implausible in this experimental setting. Conversely, priors should not force parameters toward nominal expectations when the observations support another value. Thus, if the experimental responses support an LDR of 4.90% cell-growth inhibition, the prior should not constrain it to the theoretical value of 0%.

Prior distributions are commonly described according to their informativeness. More informative priors concentrate probability over narrower parameter ranges, whereas weakly informative priors retain broader support while excluding implausible values. Prior selection necessarily incorporates assumptions about the experimental context, but these assumptions should remain transparent and should not dominate well-supported information from the observed responses. In our previous study, we showed that overly informative priors can unduly constrain the inference and yield posterior distributions that are poorly supported by the experimental data.^9^

Uniform distributions are sometimes used to represent weak prior information, but they are not inherently non-informative because their lower and upper bounds can strongly influence the inference.^11,12^ We therefore used normall, log-normal, and mixture priors that provided broad support over experimentally plausible parameter ranges. These distributions allowed us to avoid imposing hard bounds that would need to accommodate experiments spanning different concentration ranges and units, particularly for $IC_{50}$. The priors could nevertheless be adjusted for a more specific experimental context when appropriate. A previous study of prior selection for dose-response analysis obtained similar results using gamma, uniform, and normal priors and identified overly narrow priors as the main limitation.^13^ We therefore prioritized sufficiently broad prior distributions over any particular distribution family.

When defining the priors, we considered the following:

$\boldsymbol{I}\boldsymbol{C}_{\boldsymbol{50}}$: Because $IC_{50}$ is expressed on the $\log_{10}$-concentration scale, it can take both negative and positive values. We therefore used a broad normal prior that accommodates a wide range of experimentally plausible concentrations.

**LDR**: The LDR prior is relatively informative because this parameter represents the response at low or absent compound exposure and is generally expected to remain near the basal response. Moreover, LDR is typically supported by measurements at the lowest tested concentrations, even when the high-dose portion of the response is incomplete.

**HDR**: In contrast, the HDR prior is broad because the expected response at high doses varies substantially among experiments. Its mixture distribution was elicited from bioinformaticians, medicinal chemists, and biologists to assign substantial probability to strongly responsive experiments while retaining support for partially responsive and unresponsive experiments.

**Slope** and **σ**: We used log-normal priors for the slope and the standard deviation of the likelihood to constrain both parameters to positive values.^5^

The priors were defined through prior elicitation.^14^ We calibrated their locations and widths to reflect the expectations of domain experts while maintaining broad support over experimentally plausible parameter values. The resulting prior distributions encompassed the parameter ranges relevant to at least 95% of the more than 420,000 experiments analyzed (shaded regions in Fig. 1B-C). Consequently, the priors exert greater influence when the responses do not support precise inference of a parameter, such as HDR when no high-dose plateau is observed, while well-supported parameters are primarily determined by the likelihood. Priors with comparable support but different distributional shapes would therefore be expected to yield similar conclusions, with the largest differences occurring for incomplete and unresponsive experiments.

We assessed the influence of the priors using the random-pair control analysis (Supp. Fig. 6) and a sensitivity analysis comparing alternative prior specifications (Supp. Fig. 10). In the random-pair control, orientation-averaged Pearson and Spearman correlations were approximately zero for both Levenberg-Marquardt estimates and BiDRA posterior quantile-specific summaries. Thus, although shared priors can influence individual uncertainty-dominated posteriors, they did not by themselves induce positive correlations between unrelated experiments. In Supplementary Figure 10, posterior dose-response curves obtained using the elicited priors remained compatible with the observed responses across different levels of response completeness and variability. As the number of informative observations decreased, the posterior range of plausible curves broadened while remaining concentrated on experimentally plausible response values. By contrast, very broad uniform priors permitted greater posterior support for extreme curves in weakly identified cases, whereas Levenberg-Marquardt point estimates sometimes generated curves extending to extreme response values. These analyses illustrate how the elicited priors regularize poorly supported parameters without preventing the model from representing response patterns supported by the data.

Supp. Note 10. Estimating efficiency metrics from incomplete response sets

Given a set of responses, the Levenberg-Marquardt algorithm estimates θ by minimizing the sum of squared residuals between the observed and predicted responses. Minimizing this quantity is equivalent to minimizing the root mean square error for a fixed number of observations. The iterative optimization terminates when a convergence criterion is reached or when the maximum number of iterations is exceeded.

Let us consider an extreme hypothetical case in which the response set contains a single measurement. The algorithm may identify a curve that passes through this observation and therefore produces a residual close to zero. However, infinitely many log-logistic curves can pass through a single point. The observation constrains the expected response at that concentration but does not separately identify the four efficiency metrics. The resulting point estimates will therefore depend strongly on the initial parameter values and optimization path and may fall in extreme or experimentally implausible regions. We approximate this situation in Supplementary Figure 10B-C by retaining only two concentrations from synthetic datasets. Regardless of response variance, the Levenberg-Marquardt estimates span broad ranges, frequently extend beyond the experimental concentration or response ranges, and generate dose-response curves with varied shapes and orientations.

Supplementary Figure 10 illustrates this behavior across different levels of response completeness. When the observed responses cover both plateaus and the transition between them (12 to 9 responses; Supp. Fig. 10B-C), the estimated efficiency metrics remain close to their known values in the synthetic data. Their dispersion reflects response variability, with broader distributions for the $\sigma=10.0$ dataset than for the $\sigma=1.0$ dataset. When the high-dose plateau is only partially observed, LDR and IC_50_ remain comparatively well supported in these simulations (Supp. Fig. 10D). By contrast, HDR is weakly supported, and slope inference is also affected because the steepness of the transition depends on the locations of both plateaus. The quality of the resulting HDR estimates depends on both response completeness and variability. Estimates obtained from eight-response sets are more concentrated and closer to the known value than those obtained from seven-response sets (Supp. Fig. 10B–C). At the same level of completeness, HDR estimates are also closer to the known value of $HDR=0.0$ when $\sigma=1.0$ than when $\sigma=10.0$ (Supp. Fig. 10E).

With more severe incompleteness (six to two responses; Supp. Fig. 10B-C), the estimation problem approaches the underdetermined scenario described above. When the retained responses vary across concentrations rather than forming a flat profile, Levenberg-Marquardt frequently places IC_50_ near the highest tested concentration. In other cases, the inferred curve orientation is reversed and IC_50_ falls below the lowest tested concentration. These solutions can fit the available responses closely even though the corresponding efficiency metrics are poorly supported by the data.

Levenberg-Marquardt estimates efficiency metrics solely from the observed responses and returns a specific point estimate even when the data do not support precise metric inference. In such cases, multiple parameter combinations can produce similarly small residuals, and the selected estimates may be strongly influenced by initialization and the optimization trajectory. Consequently, a close fit to an incomplete response set does not imply that the resulting efficiency metrics are well supported.

# Supplementary Figures

## Supp. Figure 1. Orientation-averaged Spearman rank correlations of efficiency metrics between biological replicates

**Supplementary Figure 1***. Orientation-averaged Spearman rank correlations of efficiency metrics between biological replicates*. Orientation-averaged Spearman rank correlation coefficients are shown across efficiency metrics, datasets, and response-completeness groups. Rows correspond to efficiency metrics, dataset groups correspond to Gray, gCSI, and CTRPv2, and columns correspond to all replicate pairs, complete pairs (both experiments with response SD ≥ 20), and incomplete pairs (both experiments with response SD < 20). Gray bars represent Levenberg-Marquardt point estimates. Colored bars represent the 15 BiDRA posterior quantile-specific summaries, ordered from the lowest quantile (blue) to the highest quantile (red). Dashed horizontal lines indicate the correlation obtained from the BiDRA posterior median.

## Supp. Figure 2. Replicate-wise comparison of low-dose response estimates

**Supplementary Figure 2**. *Replicate-wise comparison of low-dose response estimates.* LDR values are compared between biological replicates using Levenberg-Marquardt point estimates (left three columns) and BiDRA posterior distributions (right three columns). Rows correspond to Gray, gCSI, and CTRPv2. Within each method, columns show all replicate pairs, complete pairs (both experiments with response SD ≥ 20), and incomplete pairs (both experiments with response SD < 20). $N$ indicates the total number of replicate pairs in each group, whereas $n$ indicates the number represented after excluding pairs without valid metric values. Dashed red lines indicate LDR = 100%. Across datasets and response-completeness groups, LDR values are concentrated near 100%, reflecting the limited variability of the basal response.

## Supp. Figure 3. Comparison of experimental concentration ranges between biological replicates in gCSI

**Supplementary Figure 3**. *Comparison of experimental concentration ranges between biological replicates in gCSI.* **(A)** Comparison of the minimum and maximum $\log_{10}$-transformed experimental concentrations between the two experiments of each replicate pair. Pairs with identical minimum or maximum concentrations fall on the diagonal ($x=y$). Point shading indicates the number of replicate pairs represented at each coordinate, as shown by the scale. **(B)** Distributions of the minimum and maximum experimental concentrations among all experiments belonging to a replicate pair ($n=4,088$). **(C)** Shared experimental concentration range for each biological replicate pair. Each horizontal segment spans the overlap between the concentration ranges of the two experiments.

## Supp. Figure 4. Replicate-wise comparison of Area Above the Curve (AAC)

**Supplementary Figure 4**. *Replicate-wise comparison of Area Above the Curve (AAC).* **(A)** AAC values are compared between biological replicates in gCSI using Levenberg-Marquardt point estimates and BiDRA posterior medians. AAC was calculated over the experimental concentration range shared by the two experiments in each replicate pair, using LDR as the baseline. Columns correspond to all replicate pairs, complete pairs (both experiments with response SD ≥ 20), and incomplete pairs (both experiments with response SD < 20). Hexbin shading indicates the number of replicate pairs represented. **(B)** Orientation-averaged Pearson and Spearman rank correlation coefficients for replicate-wise AAC comparisons across Gray, gCSI, and CTRPv2 and the three response-completeness groups. Values are reported separately for Levenberg-Marquardt point estimates and BiDRA posterior medians. The number of replicate pairs included in each comparison is indicated in parentheses by $n$.

## Supp. Figure 5. Special cases of AAC calculation for Levenberg-Marquardt and BiDRA

**Supplementary Figure 5**. *Special cases of AAC calculation for Levenberg-Marquardt and BiDRA.* (**A**) Examples of experiments for which AAC calculation from Levenberg-Marquardt point estimates resulted in *NaN* or *Inf*. These cases arise when Levenberg-Marquardt returns poorly supported efficiency metrics, often with extreme values, for response sets characteristic of unresponsive or weakly identified curves. Such values are highlighted in red in each panel. The corresponding fitted dose-response curve is shown in black. Dashed vertical lines indicate the experimental concentration range used for AAC calculation, defined as the concentration range shared with the corresponding biological replicate (not shown). **(B)** Left: Comparison of LDR${}_{i}{}$ and HDR${}_{i}{}$ values for BiDRA posterior draws resulting in negative AAC values. Across all experiments belonging to a biological replicate pair ($n=4,088$), 1,676 had at least one posterior draw yielding a negative AAC. A total of 142,465 posterior draws are represented. Right: Relationship between negative AAC values and response completeness, measured by the response standard deviation. Experiments were classified as complete when response SD ≥ 20 and incomplete when response SD < 20. **(C)** Distribution, for each experiment represented in panel **B**, of the proportion of posterior draws yielding a negative AAC. The upper panel corresponds to complete experiments ($n=68$), and the lower panel corresponds to incomplete experiments ($n=1,608$).

## Supp. Figure 6. Spearman’s rank correlations for control experiments

**Supplementary Figure 6**. *Permutation control for replicate-wise correlations of efficiency metric representations*. The first replicate of each biological pair was retained, while second replicates were randomly permuted across pairs, preserving the number and marginal composition of the replicate sets while disrupting the original (cell-line, compound) correspondence. Orientation-averaged Pearson **(A)** and Spearman rank **(B)** correlation coefficients are shown across efficiency metrics (rows), datasets (column triplets), and randomized-pair groupings (columns). Gray bars represent Levenberg-Marquardt point estimates, whereas colored bars represent the 15 BiDRA posterior quantile-specific summaries, ordered from lower to upper quantiles. Dashed horizontal lines indicate the coefficient obtained from the BiDRA posterior median, and solid horizontal lines indicate zero correlation. Pair groupings were defined using response standard deviation (SD) as a proxy for curve completeness. Complete Pairs contain two experiments with SD ≥ 20, whereas Incomplete Pairs contain two experiments with SD < 20.

## Supp. Figure 7. Comparison of IC50 and HDR estimates and their posterior for Gray and CTRPv2

**Supplementary Figure 7**. *Comparison of IC_50_ and HDR estimates and their posterior for Gray and CTRPv2*. **(A)** Analysis of the Gray dataset. **(B)** Analysis for the CTRPv2 dataset. In each dataset, the **two upper-left** panels compare response standard deviation (SD) with Levenberg-Marquardt IC_50_ point estimates (left) and BiDRA IC_50_ posterior distributions (right). The red horizontal line indicates the threshold used to distinguish complete (SD ≥ 20) from incomplete or unresponsive (SD < 20) response sets. The interval denoted by $D$ corresponds to the central experimental concentration range, defined from the empirical distribution of tested concentrations across experiments; the black vertical lines indicate its bounds. All experiments were included in the analysis ($N$), whereas only a subset is displayed in the Levenberg-Marquardt point-estimate panels ($n$) to contain the horizontal axis of the plot. The **lower-left** panel shows, for each experiment, the posterior probability that IC_50_ lies outside $D$, $Pr\left( IC_{50}\notin D \right)$, as a function of response SD. The adjacent boxplots summarize SD values after classifying Levenberg-Marquardt IC_50_ estimates as inside or outside $D$, shown for all experiments and for the subset in which the Levenberg-Marquardt algorithm converged. The **right** panel compares Levenberg-Marquardt HDR point estimates with ΔHDR, a measure of BiDRA posterior width for HDR. Together, these panels illustrate that incomplete or unresponsive response sets are associated with weakly supported IC_50_ estimates and broader HDR posteriors.

## Supp. Figure 8. Relationship between response completeness, HDR estimates, and HDR posterior width

**Supplementary Figure 8**. *Relationship between response completeness, HDR estimates, and HDR posterior width*. Response completeness was assessed using the standard deviation (SD) of the observed responses. Experiments with SD ≥ 20 were classified as complete, whereas those with SD<20 were classified as incomplete or unresponsive. For each dataset, the **left** panel compares response SD with Levenberg-Marquardt HDR point estimates, and the **right** panel compares response SD with BiDRA HDR posterior width (ΔHDR), defined as the difference between the upper and lower bounds of the 95% credible interval. The red horizontal line indicates the SD = 20 completeness threshold. Incomplete or unresponsive experiments frequently yielded extreme Levenberg-Marquardt HDR estimates and broad BiDRA HDR posteriors, indicating that the data did not support precise HDR inference. The number of experiments represented in each panel is denoted by $n$, and the total number of experiments in each dataset is denoted by $N$.

## Supp. Figure 9. Distribution of replicate structures within and across datasets

**Supplementary Figure 9**. *Distribution of replicate structures within and across datasets*. **(A)** Number of (cell-line, compound) combinations represented by three or more experiments within each dataset, stratified by the total number of experiments per combination. Colors are dataset specific. **(B)** The left table reports the number of (cell-line, compound) combinations according to their within-dataset replicate structure: singletons (R = 1), combinations represented by exactly two experiments (R = 2), and multi-replicate combinations represented by at least three experiments (R ≥ 3). The right table reports the number of within-dataset singletons corresponding to the same (cell-line, compound) combination in each pair of datasets. Blue-shaded cells indicate the total number of singletons in each dataset.

## Supp. Figure 10. Effects of response-set incompleteness and prior specification on dose-response inference

**Supplementary Figure 10**. *Effects of response-set incompleteness and prior specification on dose-response inference.* **(A)** Synthetic response datasets used in panels B–E. Twelve response values (red points) were generated from a reference dose–response curve with $LDR=100.0$, $HDR=0.0$, $\log_{10} \left( IC_{50} \right)=0.0$, and $slope=1.0$ (red dashed curve) by adding Gaussian noise with $\sigma=1.0$ or $\sigma=10.0$. This procedure was repeated 100 times, and the resulting response distributions are shown as black violin plots. **(B)** Effect of progressively removing high-dose response values from the low-variability synthetic datasets ($\sigma=1.0$). **(C)** Corresponding analysis for the high-variability synthetic datasets ($\sigma=10.0$). Rows correspond to Levenberg-Marquardt estimation (top), Bayesian inference using the BiDRA priors (middle), and Bayesian inference using broad uniform priors (bottom). Columns indicate the number of retained responses. Starting from the complete 12-response set, measurements were removed sequentially from the highest concentration (red points). The true LDR, HDR, and log_10_ IC_50_ values are indicated by red dashed lines. Each method was applied independently to all 100 simulated response sets. For Levenberg-Marquardt, gray curves were generated from the estimated efficiency metrics. For each Bayesian model, one posterior draw from each simulated response set was used to generate a gray curve, yielding 100 curves per method and condition. The marginal distributions of log_10_ IC_50_, HDR, and LDR are shown along the upper and right margins. Colored curves for the eight-response condition correspond to the distributions summarized in panels **D** and **E**. **(D)** Differences between the true and estimated or inferred log_10_ IC_50_ values for the three methods when eight responses were retained and the high-dose plateau was no longer observed. Results are shown for both noise levels. **(E)** Differences between the true and estimated or inferred HDR values under the same conditions. The Δ distributions represent deviations from the known values HDR = 0.0 and log_10_ IC_50_ = 0.0. Distributions were truncated for visualization. Colors identify the inference method and are consistent with panels **B** and **C**.

## Supp. Figure 11. Comparison of the computational efficiency of Levenberg-Marquardt and BiDRA

**Supplementary Figure 11**. *Comparison of the computational efficiency of Levenberg-Marquardt and BiDRA.* Execution times were measured on an AMD EPYC 7763 processor using four threads. For each response-set size and noise level (σ = 1.0 or σ = 10.0) considered in Supplementary Figure 10, we recorded execution times for 10 randomly selected synthetic response sets. For Levenberg-Marquardt, we measured the execution time of the ‘curve_fit()’ function from LsqFit.jl. For BiDRA, we measured the execution time of Turing.jl’s ‘sample()’ function, with progress reporting disabled and the four MCMC chains executed using ‘MCMCThreads()’. The upper and middle rows show execution times for BiDRA and Levenberg-Marquardt, respectively. The lower row shows the ratio of BiDRA to Levenberg-Marquardt execution times. Reported times include only efficiency-metric estimation or inference and exclude subsequent data processing, analysis, and plotting.

# References

1.	Feizi, N. *et al.* PharmacoDB 2.0: improving scalability and transparency of in vitro pharmacogenomics analysis. *Nucleic Acids Res* **50**, D1348–D1357 (2022).

2.	Mammoliti, A. *et al.* Orchestrating and sharing large multimodal data for transparent and reproducible research. *Nat Commun* **12**, 5797 (2021).

3.	Smirnov, P. *et al.* PharmacoGx: an R package for analysis of large pharmacogenomic datasets. *Bioinformatics* **32**, 1244–1246 (2016).

4.	DeLean, A., Munson, P. J. & Rodbard, D. Simultaneous analysis of families of sigmoidal curves: application to bioassay, radioligand assay, and physiological dose-response curves. *American Journal of Physiology-Endocrinology and Metabolism* **235**, E97-102 (1978).

5.	Scheff, J. D., Almon, R. R., DuBois, D. C., Jusko, W. J. & Androulakis, I. P. Assessment of Pharmacologic Area Under the Curve When Baselines are Variable. *Pharmaceutical Research* **28**, 1081–1089 (2011).

6.	Yadav, B. *et al.* Quantitative scoring of differential drug sensitivity for individually optimized anticancer therapies. *Scientific Reports* **4**, 5193 (2014).

7.	Levenberg, K. A method for the solution of certain non-linear problems in least squares. *Quarterly of Applied Mathematics* **2**, 164–168 (1944).

8.	Marquardt, D. W. An Algorithm for Least-Squares Estimation of Nonlinear Parameters. *Journal of the Society for Industrial and Applied Mathematics* **11**, 431–441 (1963).

9.	Labelle, C., Marinier, A. & Lemieux, S. Enhancing the drug discovery process: Bayesian inference for the analysis and comparison of dose–response experiments. *Bioinformatics* **35**, i464–i473 (2019).

10.	Hoffman, M. D. & Gelman, A. The No-U-turn sampler: adaptively setting path lengths in Hamiltonian Monte Carlo. *J. Mach. Learn. Res.* **15**, 1593–1623 (2014).

11.	Gelman, A. *et al.* *Bayesian Data Analysis*. (CRC Press, 2013).

12.	Lambert, B. *A Student’s Guide to Bayesian Statistics*. (SAGE Publications, 2018).

13.	Huson, L. W. & Kinnersley, N. Bayesian fitting of a logistic dose–response curve with numerically derived priors. *Pharmaceutical Statistics* **8**, 279–286 (2009).

14.	Albert, I. *et al.* Combining Expert Opinions in Prior Elicitation. *Bayesian Analysis* **7**, 503–532 (2012).
